# Corrosion Behavior and Strength of Dissimilar Bonding Material between Ti and Mg Alloys Fabricated by Spark Plasma Sintering

**DOI:** 10.3390/ma9080665

**Published:** 2016-08-06

**Authors:** Patchara Pripanapong, Shota Kariya, Tachai Luangvaranunt, Junko Umeda, Seiichiro Tsutsumi, Makoto Takahashi, Katsuyoshi Kondoh

**Affiliations:** 1 Graduate School of Engineering, Osaka University, 2-1 Yamadaoka, Suita, Osaka 565-0871, Japan; kariya-s@jwri.osaka-u.ac.jp; 2 Department of Metallurgical Engineering, Chulalongkorn University, Phayathai, Patumwan, Bangkok 10330, Thailand; tachai.l@chula.ac.th; 3 Joining and Welding Research Institute, Osaka University, 11-1 Mihogaoka, Ibaragi, Osaka 567-0047, Japan; umedaj@jwri.osaka-u.ac.jp (J.U.); tsutsumi@jwri.osaka-u.ac.jp (S.T.); makotot@jwri.osaka-u.ac.jp (M.T.); kondoh@jwri.osaka-u.ac.jp (K.K.)

**Keywords:** Ti, Mg alloys, spark plasma sintering, bonding strength, galvanic corrosion

## Abstract

Ti and solution treated Mg alloys such as AZ31B (ST), AZ61 (ST), AZ80 (ST) and AZ91 (ST) were successfully bonded at 475 °C by spark plasma sintering, which is a promising new method in welding field. The formation of Ti_3_Al intermetallic compound was found to be an important factor in controlling the bonding strength and galvanic corrosion resistance of dissimilar materials. The maximum bonding strength and bonding efficiency at 193 MPa and 96% were obtained from Ti/AZ91 (ST), in which a thick and uniform nano-level Ti_3_Al layer was observed. This sample also shows the highest galvanic corrosion resistance with a measured galvanic width and depth of 281 and 19 µm, respectively. The corrosion resistance of the matrix on Mg alloy side was controlled by its Al content. AZ91 (ST) exhibited the highest corrosion resistance considered from its corrode surface after corrosion test in Kroll’s etchant. The effect of Al content in Mg alloy on bonding strength and corrosion behavior of Ti/Mg alloy (ST) dissimilar materials is discussed in this work.

## 1. Introduction

The applications of lightweight materials are gradually increased in many industrial fields recently. Titanium is one of well-known lightweight materials, which is applied in many industrial fields such as automobile, aerospace, medical prosthesis and chemical industry. This is attributed to the advantageous properties of Ti such as high specific strength, good biocompatibility and corrosion resistance [1,2,3,4]. In order to reduce the weight of automobile and aerospace component, a new light dissimilar material between Ti and Mg alloy was fabricated in this research. The application of this light material will reduce fuel consumption and pollution emitted to the environment from vehicles. However, reports related to the bonding of Ti and Mg alloy are still scarce because of the difficulty in bonding, as no intermetallic compounds exist between Ti and Mg, referring to the binary phase diagram [5]. Transient liquid phase bonding between AZ31 (Mg alloy) and Ti64 was reported by Atieh et al. However, pure Ni coated on Ti64 surface was necessary to obtain a successful bond. The thick intermetallic compound (IMC) layer that degraded the shear strength of bonding material was formed due to the eutectic liquid phase formation during bonding [6]. Fouad studied the bonding between pure Ti and AZ31 with Al inserted sheet fabricated by hot isostatic pressing (HIP), and subsequent annealing. However, bonding components showed a poor tensile strength due to the formation of thick intermetallic layer at the Al/AZ31 interface [7]. 

The corrosion resistance, especially the galvanic corrosion of the bonding material, is another important property similar to the tensile strength as the bonding components are used under corrosive atmosphere [8,9,10]. The report related to the effect of intermetallic layer formation at the bonding interface on the galvanic corrosion resistance of the bonding material was also scarce. Funutsu et al. fabricated the carbon nanotubes (CNTs) reinforced Mg composites, and studied the galvanic corrosion of this material. They proposed that the galvanic corrosion between *α*-Mg matrix and CNTs was relieved by the concentration of Al atoms around CNTs via heat treatment. This phenomenon caused the reduction of surface potential difference (SPD) between *α*-Mg matrix and CNTs [11]. Huang et al. studied the galvanic corrosion behavior of Inconel 718 after electron beam welding. The galvanic corrosion resistance of solution-annealed Inconel 718 weld was improved by applying post-welding precipitation hardening treatment. The post-weld heat treatment caused a disappearance of the heat affected zone (HAZ), which existed between fusion zone and base metal, resulting in a decreasing of the corrosion potential difference between fusion zone and base metal [12]. Norouzi studied the effect of brazing on the corrosion resistance of clad modified Al alloys between AA4xxx/AA3xxx. The results show that the voltage potential of re-solidified matrix (clad) was increased after brazing, and the voltage potential difference between re-solidified matrix and Si needle at the bonding interface was decreased. This resulted in an improvement of the galvanic corrosion resistance [13].

In this research, the effect of intermetallic compound (IMC) layer formation on tensile strength and galvanic corrosion resistance of the dissimilar bonding materials between Ti and Mg alloys were studied. The galvanic corrosion in each dissimilar material was predicted by scanning kelvin probe force microscope (SKPFM), which is used for SPD measurement. The corrosion behavior of dissimilar materials that are different for each Mg–Al alloy was elucidated by mass loss measurement and surface profile analysis after corrosion test.

## 2. Materials and Methods

**Figure 1:**
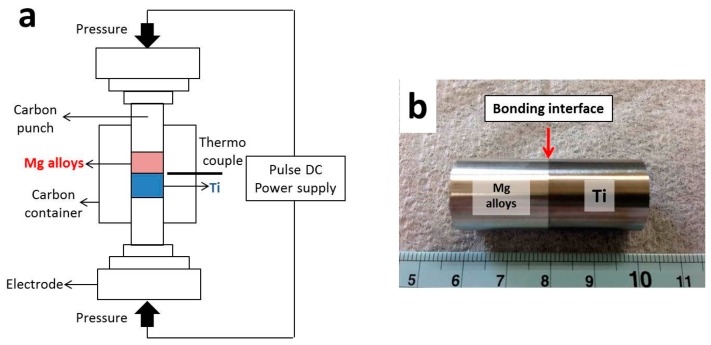
Schematic drawing of component setting in SPS chamber (**a**); and bonded sample (**b**).

Ti rod (16 mm in diameter) with purity of 99.95% and four types of cast Mg alloy rod (16 mm in diameter), AZ31B, AZ61, AZ80 and AZ91, were used in this research. All materials were purchased from Nilaco Co., Ltd. (Tokyo, Japan). The chemical composition of Ti and Mg alloys are listed in Table 1 and Table 2, respectively. All Mg alloys were solution-treated at 420 °C for 12 h in muffle furnace and subsequently quenched in water before surface preparation. Pure Ti and Mg alloys rods were cut into 20 mm height, and their surfaces were prepared before bonding process. The surface of Ti was ground by SiC emery paper until 2000# and polished with 0.05 µm Al_2_O_3_ colloidal. On the other hand, the surfaces of Mg alloys were ground by SiC emery paper until 2000# and polished with 0.25 µm diamond paste. The polished Ti and Mg alloy rod were inserted in a carbon container with an inner diameter of 16 mm, and the carbon punches were placed at both ends of the sample. Ti and Mg alloys were bonded together by a spark plasma sintering (SPS) machine (SYNTECH Co. SPS103S, Sinterland Inc., Niigata, Japan), in which a sample was heated by a flow of current through an electrode and carbon container. The thermocouple was inserted in carbon container wall with a distance far from a sample of 1 mm to provide a precise temperature measurement. The schematic drawing of the component setting in SPS chamber and bonded sample are shown in Figure 1a,b, respectively. Ti and Mg alloys were bonded at 400 and 475 °C for 1 h under a bonding pressure of 10 MPa in a vacuum atmosphere, and subsequently cooled in SPS chamber. For microstructure observation, samples were cut at the bonding interface, and their surfaces were prepared by the same method as applied for preparing Mg alloy surface followed by etching with picric acid. Microstructure observations were performed by scanning electron microscope (JEOL JSM6500F, JEOL Ltd., Tokyo, Japan) and transmission electron microscope (JEOL JEM-2010, JEOL Ltd., Tokyo, Japan). Three tensile specimens were machined from a bonding sample with a gauge length and diameter of 20 and 3 mm, respectively. Tensile test was performed at room temperature under testing speed of 0.05 mm/min, and load direction is normal to the bonding interface. The bonding efficiency was simply calculated by Equation (1).

(1) Bonding efficiency=(σb/σp)×100

where *σ*_b_ = Bonding strength of bonding sample (MPa); and *σ*_p_ = Tensile strength of parent Mg alloys (MPa).

The surface potential of Ti and Mg alloys and surface potential difference (SPD) at the bonding interface were measured by scanning kelvin probe force microscope or SKPFM (WET-SPM, SHIMADZU, Shimadzu Co., Kyoto, Japan) before corrosion test. The scanning area was 30 µm × 30 µm and the scan line at the bonding interface was 10 µm. The surface potential that measured from SKPFM is an absolute value with a platinum reference electrode. The bonding samples were cut to a size of 8 mm × 8 mm × 4 mm and immersed in Kroll’s etchant (HF:HNO_3_:H_2_O = 1:5:100) at room temperature. The Kroll’s etchant was selected for corrosion testing because it can corrode both titanium and Mg alloy, which is suitable for studying corrosion behavior of dissimilar materials. Furthermore, the corroded surfaces of dissimilar materials were cleaned without corrosion product resulted in a precise measurement of galvanic width and depth.

The sample was immersed in Kroll’s etchant for 2 and 10 min for surface profile analysis and mass loss measurement, respectively. The surface profile analysis after corrosion test was performed by surface analysis microscope (KEYENCE VR-3100, Keyence Co., Osaka, Japan). The galvanic width and depth were measured to evaluate a severity of galvanic corrosion. The mass of dissimilar materials were measured before and after corrosion test and simply calculated to percent of mass loss to study the effect of aluminum content in Mg alloy on corrosion resistance of dissimilar materials. The mass loss of dissimilar materials was also compared to a mass loss of Mg alloy parent metal (sample size of 8 mm × 4 mm × 4 mm) to confirm the existence and severity of galvanic corrosion.

## 3. Results and Discussion

### 3.1. Microstructure Analysis

**Figure 2:**
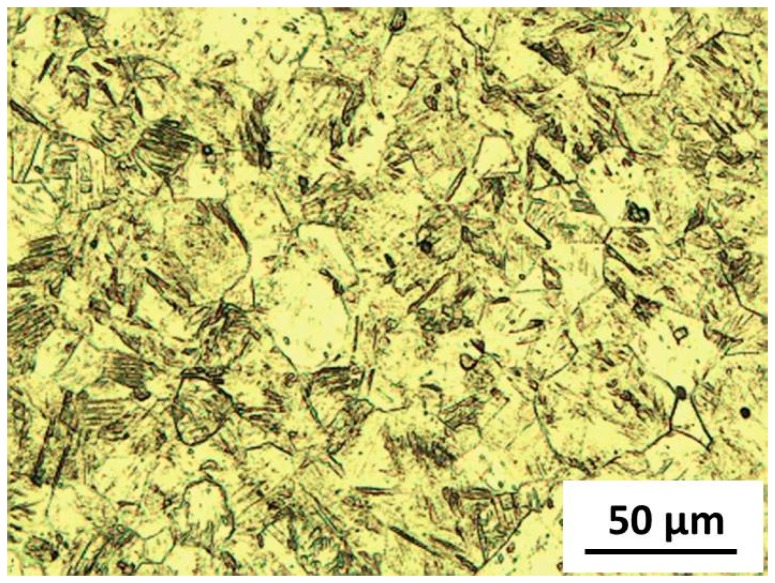
Microstructure of pure Ti observed by optical microscope.

**Figure 3:**
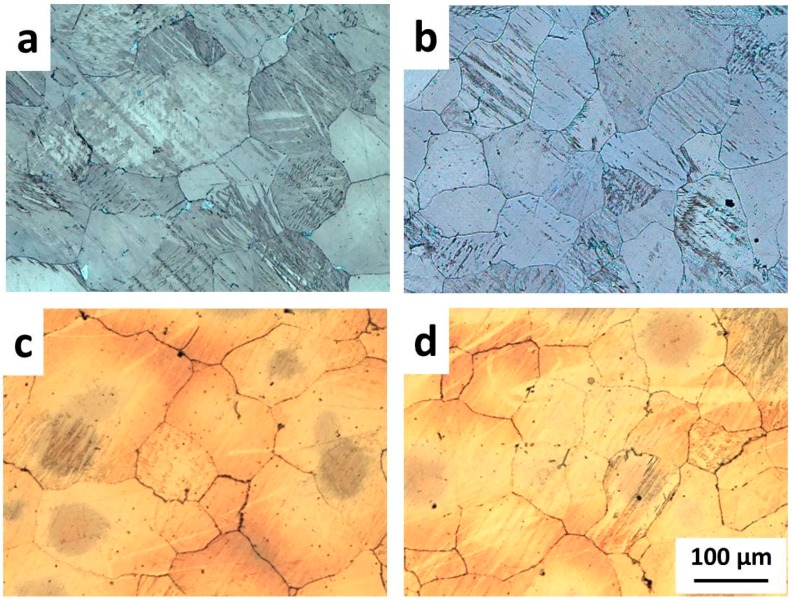
Microstructures of Mg alloys after solution treatment at 420 °C for 12 h and being quenched in water: (**a**) AZ31B; (**b**) AZ61; (**c**) AZ80; and (**d**) AZ91.

The optical microstructure of pure Ti applied in this research is shown in Figure 2. The pure Ti consisted of *α*-Ti matrix with no other phases, and its grain size was approximately 30 µm. Figure 3a–d shows microstructures of Mg alloys after solution treatment at 420 °C for 12 h and subsequent quenching in water. All Mg alloys show a similar microstructure and grain size, in which only Mg matrix and twin was observed. The purpose of solution treatment was to dissolve the brittle *β*-Mg (Mg_17_Al_12_) particles, which inhibited bonding between Ti and Mg alloy into the matrix, and to create a uniform Al distribution on the bonding surface of Mg alloys [14]. Hereafter, the solution-treated Mg alloy was named as Mg alloy (ST).

**Figure 4:**
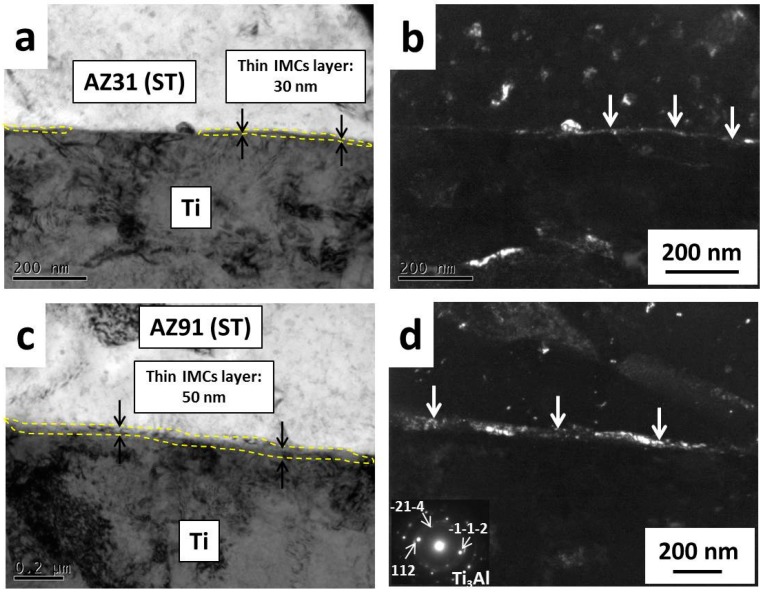
Bonding interface of Ti/AZ31B (ST) and Ti/AZ91 (ST) bonded at 475 °C for 1 h: (**a**) bright field Ti/AZ31B (ST); (**b**) dark field Ti/AZ31B (ST); (**c**) bright field Ti/AZ91 (ST); and (**d**) dark field Ti/AZ91 (ST).

Figure 4 shows the bonding interfaces of Ti bonded to AZ31B (ST) or Ti/AZ31B (ST) and Ti/AZ91 (ST) at 475 °C for 1 h under bonding pressure of 10 MPa. Figure 4a shows the bonding interface of Ti/AZ31B (ST) which an existence of discontinuous Ti3Al IMC layer with a thickness of 30 nm was observed, and confirmed by diffraction pattern in Figure 4d. This IMC layer was also detected in a dark field image, in which the IMC layer is brighter than the other areas (Figure 4b). The bonding interface characteristic of Ti/AZ61 (ST) observed by TEM was similar to Ti/AZ31B (ST) but the thickness of the IMC layer slightly increased to 36 nm. Figure 4c shows the bonding interface of Ti/AZ91 (ST) bonded at 475 °C for 1 h under an applied pressure of 10 MPa. The bright field image shows the existence of a Ti_3_Al IMC layer at the bonding interface with a thickness of 50 nm (Figure 4c), which was confirmed by a dark field image and diffraction pattern (Figure 4d). This intermetallic compound, which was observed at the bonding interface, was a continuous layer, implying that a reaction between Al in Mg alloy and Ti at the bonding interface was simpler and more uniform compared to Ti/AZ31B (ST) and Ti/AZ61 (ST). This was explained by the increased content of Al in Mg alloy: more Al atoms cause a strong reaction between Ti and Al and result in an increased thickness of the IMC layer. For Ti/AZ80 (ST), the thickness of Ti_3_Al IMC layer slightly decreased to 47 nm compared to Ti/AZ91 (ST). The dark areas in Figure 4b,c on both Ti and Mg alloy matrix are high dislocation density areas, which were affected by a bonding pressure that causes a plastic deformation in both materials. The plastic deformation facilitated the two surfaces to perfectly contact, and this corresponded well to the TEM images that no cavities were observed on the bonding interface [15]. The Al diffusion layer was also observed on the bonding interface in each bonding material by TEM-EDS and its thickness was similar to the thickness of the IMC layer.

### 3.2. Bonding Strength Evaluation

**Figure 5:**
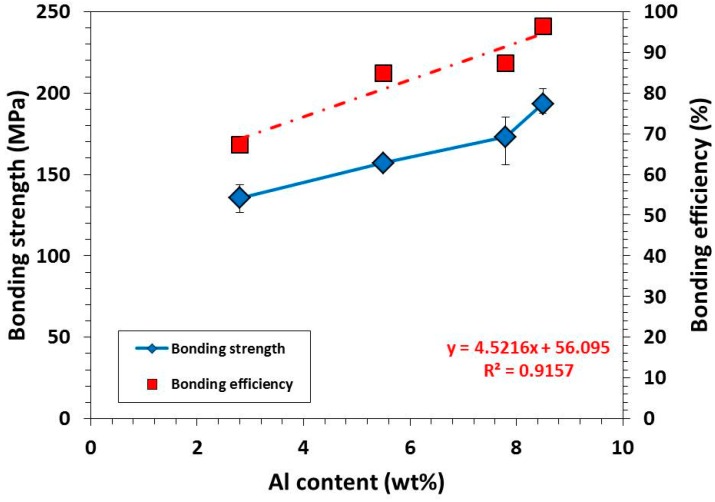
Effect of Al content in Mg alloy on bonding strength and bonding efficiency.

Figure 5 shows bonding strength and bonding efficiency of Ti/Mg alloy dissimilar materials bonded at 475 °C for 1 h under bonding pressure of 10 MPa. Ti/AZ31B (ST) shows the lowest bonding strength among the bonding materials with the value of 136 ± 9 MPa. This was explained by a degree of reaction between Ti and Al that this material shows the thinnest Al diffusion and Ti_3_Al layer of 30 nm (Figure 4a). The increasing of Al content in Ti/AZ61 (ST) compared to Ti/AZ31B (ST) resulted in an increasing of bonding strength from 136 ± 9 to 157 ± 3 MPa. This was attributed to an increase in the thickness of Al diffusion and Ti_3_Al layer from 30 to 36 nm. However, the characteristic of Ti_3_Al layer was similar to Ti/AZ31B (ST), as it was a discrete layer. For Ti/AZ80 (ST), the bonding strength was increased to 173 ± 15 MPa with an increasing in thickness of Ti_3_Al layer to 47 nm. Another reason that contributed to this improvement was a change in a characteristic of Ti_3_Al layer from a discrete to continuous layer (Figure 4b). Ti/AZ91 (ST) exhibited the highest bonding strength of 193 ± 8 MPa, which was expected from its bonding interface characteristic that a formation of a continuous and uniform Ti_3_Al layer with a thickness of 50 nm was observed and confirmed by dark field image (Figure 4d). All bonding samples were fractured on Mg alloy side near the bonding interface, and the fracture direction was perpendicular to tensile direction.

The bonding efficiency shown in Figure 5 can be simply calculated using Equation (1). The bonding efficiency indicated the compatibility between two materials when it bonded together. The result shows that the bonding efficiency was directly related to aluminum content in Mg alloy. It was gradually increased from 67% to 96% when Al content in Mg alloy was increased. The bonding efficiency of Ti/AZ91 (ST) was very satisfying: the bonding strength almost reached the tensile strength of AZ91 (ST) parent metal. These results emphasized the importance of Al diffusion and Ti_3_Al layer formation to the improvement of bonding strength.

### 3.3. Surface Potential Measurement at Bonding Interface of Dissimilar Materials

**Figure 6:**
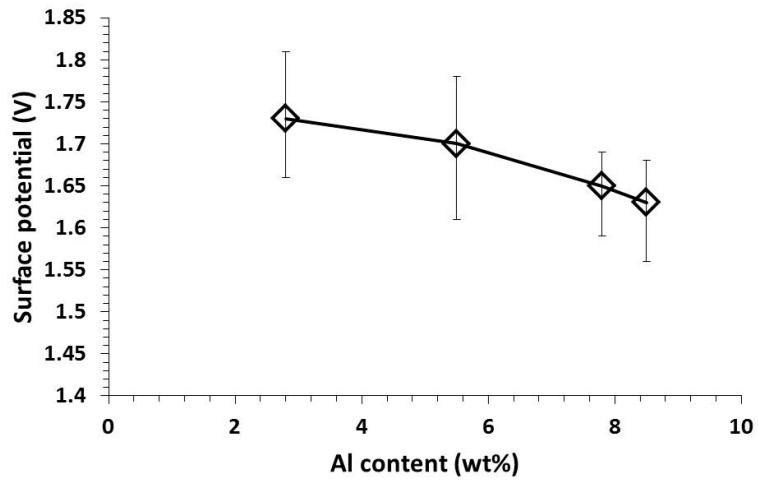
Effect of Al content on surface potential of Mg alloys.

Figure 6 shows a surface potential of Mg alloys after solution treated at 420 °C for 12 h measured by SKPFM. These surface potentials were an average value measured from five different positions. AZ31B (ST) and AZ61 (ST) show a high surface potential of 1.73 ± 0.08 and 1.70 ± 0.09 V, respectively. According to the surface potential measured from SKPFM, which is an absolute value, the material that possesses the higher surface potential is more susceptible to the corrosion compared to the lower surface potential. On the other hand, AZ80 shows a lower surface potential compared to AZ31B (ST) and AZ61 (ST) with a value of 1.65 ± 0.05 V. The increment in Al content in Mg alloy was effective in decreasing in surface potential, which was attributed to a formation of stable Mg–Al–O film on Mg alloy surface [16,17]. AZ91 (ST) shows a surface potential of 1.63 ± 0.06 V, which is the lowest among Mg alloys in this research due to its highest Al content. According to this result, AZ91 (ST) should possess the highest corrosion resistance among Mg alloys. The surface potential of pure Ti was also measured from five different positions, and it shows a value of 0.78 ± 0.03 V. This implied that pure Ti was much less susceptible to corrosion compared to Mg alloys because of the formation of a TiO_2_ surface film that was very effective at preventing corrosion.

**Figure 7:**
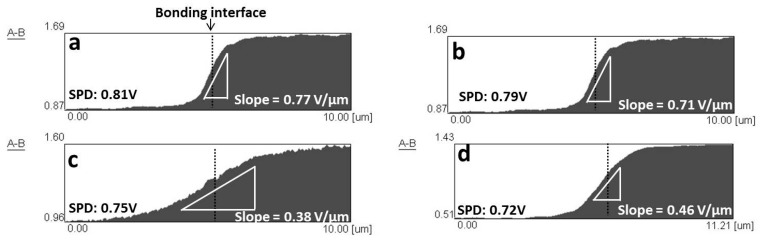
Changes in surface potential measured across the bonding interface of: (**a**) Ti/AZ31B (ST); (**b**) Ti/AZ61 (ST); (**c**) Ti/AZ80 (ST); and (**d**) Ti/AZ91 (ST) bonded at 475 °C for 1 h.

Figure 7 shows the surface potential differences at the bonding interface of Ti/Mg alloy bonding materials bonded at 475 °C for 1 h before corrosion test. From the surface potential curves, the potential gradients were measured at the bonding interface (at dot line position) in all bonding materials from three different positions. This potential gradient referred to a rate of surface potential change at the bonding interface because an existence of interaction or diffusion layer [13]. The existence of the interaction or diffusion layer will prevent a large and sudden surface potential change at the bonding interface, resulting in a reduction of the galvanic corrosion damages. For Ti/AZ31B (ST) dissimilar material, SPD between Ti and AZ31B (ST) was measured to be 0.81 V (Figure 7a). This SPD value was considerably large, and may result in severe galvanic corrosion at the bonding interface. The calculated slope, which represented a potential gradient of Ti/AZ31B (ST), was 0.77 V/µm. This value was rather high because of sudden change in surface potential at the bonding interface, and a poor galvanic corrosion resistance of this dissimilar material was predicted. The sudden change in surface potential at the bonding interface of Ti/AZ31B (ST) was attributed to a poor reaction between Ti and Al, as a thin Al diffusion layer of 30 nm was observed with the formation of thin Ti_3_Al layer in some areas (Figure 4a). Ti/AZ61 (ST) also shows a large SPD and potential gradient between Ti and Mg alloy similar to Ti/AZ31B (ST) (Figure 7b). This was explained by a similar characteristic at the bonding interface between Ti/AZ31B (ST) and Ti/AZ61 (ST): a thin Al diffusion and Ti_3_Al layer were observed. In the case of Ti/AZ80 (ST), the measured SPD value was 0.75 V (Figure 7c). This indicated that the galvanic corrosion resistance of Ti/AZ80 (ST) was superior to Ti/AZ31B (ST) and Ti/AZ61 (ST) [18]. The potential gradient of Ti/AZ80 (ST) was also much lower than Ti/AZ31B (ST) and Ti/AZ61 (ST) with a value of 0.38 V/µm. The gradual change in surface potential at the bonding interface of Ti/AZ80 (ST) confirmed that this material possessed a good galvanic corrosion resistance. The SPD and potential gradient of Ti/AZ91 (ST) were similar to that of Ti/AZ80 (ST) with a value of 0.77 V and 0.46 V/µm, respectively (Figure 7d). These results suggested that Ti/AZ80 (ST) and Ti/AZ91 (ST) should possess similar galvanic corrosion resistances.

### 3.4. Corrosion Behavior Analysis of Dissimilar Materials

**Figure 8:**
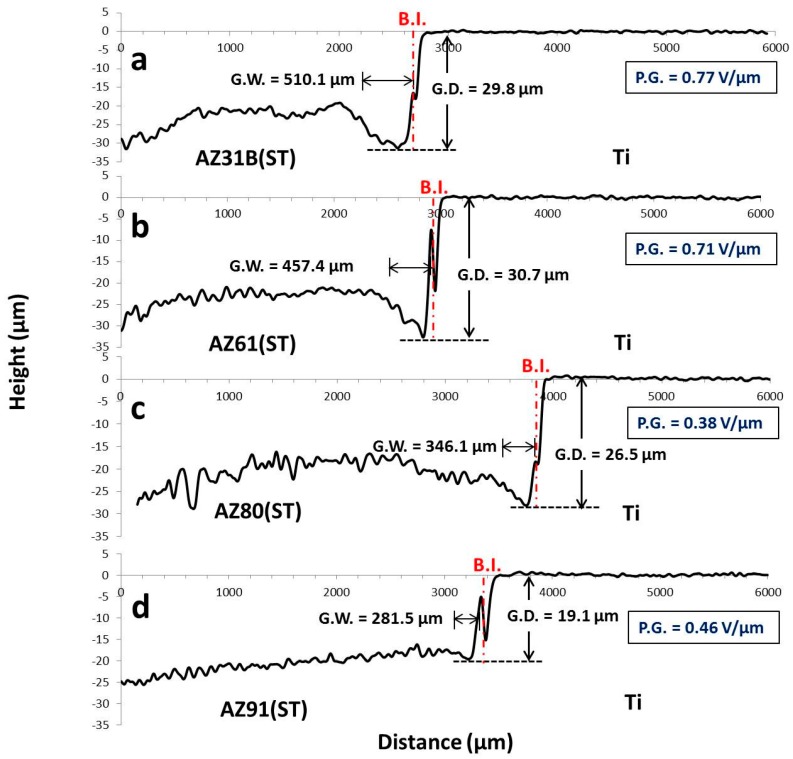
Line profile analysis at the bonding interfaces after corrosion test in Kroll’s etchant: (**a**) Ti/AZ31B (ST); (**b**) Ti/AZ61 (ST); (**c**) Ti/AZ80 (ST); and (**d**) Ti/AZ91 (ST).

Figure 8 shows the line profiles of Ti/Mg alloy (ST) dissimilar materials at the bonding interface after corrosion test in Kroll’s etchant. From these results, a galvanic corrosion between Ti and Mg alloys (ST) was confirmed by a characteristic of the plot that the surface level of Mg alloys (ST) near the bonding interface was lower than areas far from the surface. The galvanic corrosion depth (G.D.) and galvanic corrosion width (G.W.) were measured to evaluate a degree of galvanic corrosion in each bonding sample. For Ti/AZ31B (ST), high G.W. and G.D. of 510.1 and 29.8 µm, respectively, were measured. The large gap at the bonding interface between Ti and AZ31B (ST) caused by galvanic corrosion was clearly observed (Figure 8a). The surface of Ti was not damaged after corrosion test, which confirmed that corrosion mostly occurred on Mg alloy (ST) surface. This result also corresponded well to SPD between Ti and AZ31B (ST), which exhibited the highest value among bonding materials. Ti/AZ61 (ST) showed a similar G.D. compared to Ti/AZ31B (ST) but a large decrease in G.W. with a value of 457.4 µm (Figure 8b). This was explained by an increasing of Al diffusion and Ti_3_Al layer at the bonding interface, which further reduced SPD between Ti and Mg alloys (ST) compared to Ti/AZ31B (ST). Although thickness of Ti_3_Al and Al diffusion layer was at the nano-level, it was able to inhibit the progress of galvanic corrosion from the bonding interface to further areas in Mg alloy [19]. The G.W. and G.D. were further decreased in Ti/AZ80 (ST) with values of 346.1 and 26.5 µm, respectively (Figure 8c). This referred to an improvement of galvanic corrosion resistance of Ti/AZ80 (ST) compared to Ti/AZ61 (ST) with a decrease in SPD. Ti/AZ91 (ST) exhibited the lowest G.W. and G.D. with a value of 281.5 and 19.1µm, respectively (Figure 8d). The gap between Ti and Mg alloy (ST) was very small compared to other bonding materials. The formation of thick and continuous Ti_3_Al layer play an impotant role in the improvement of galvanic corrosion resistance in this sample.

**Figure 9:**
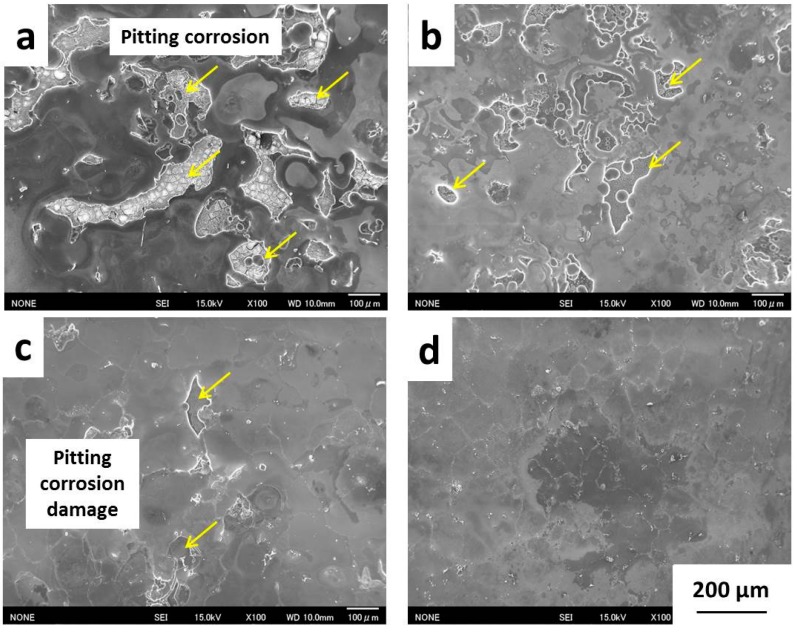
Corroded surfaces on Mg alloy (ST) side of Ti/Mg alloys (ST) dissimilar material: (**a**) AZ31B (ST); (**b**) AZ61 (ST); (**c**) AZ80 (ST); and (**d**) AZ91 (ST).

Figure 9 shows the corroded surfaces of Ti/Mg alloy (ST) bonding materials bonded at 475 °C for 1 h on Mg alloy (ST) side. AZ31B (ST), which possessed the highest surface potential of 1.73 V, shows much severer corrosion damage compared to other bonding materials (Figure 9a). Many large pitting corrosion areas were observed throughout the corroded surface. The black area near the pitting corrosion damage was an area that suffered severe corrosion, and had a lower surface level compared to the adjacent area (grey area matrix). AZ61 (ST) possessed a lower surface potential compared to AZ31B (ST), shows less severe damage compared to AZ31B (ST), the pitting corrosion areas are smaller (Figure 9b) and the black areas mostly disappeared. This indicated that the corrosion resistance of AZ61 (ST) was clearly improved compared to AZ31B (ST). For AZ80 (ST), the amounts of small pitting corrosion areas were decreased compared to AZ61 (ST) because the surface potential of AZ80 (ST) was lower than AZ61 (ST) (Figure 9c). This indicated that the corrosion resistance of AZ80 (ST) was further improved compared to AZ61 (ST). AZ91 (ST) shows superior corrosion resistance compared to other Mg alloys because the pitting corrosion damages were barely observed on the corroded surface. However, deep corroded areas were observed in many locations because of the strong acidic characteristic of Kroll’s etchant.

**Figure 10:**
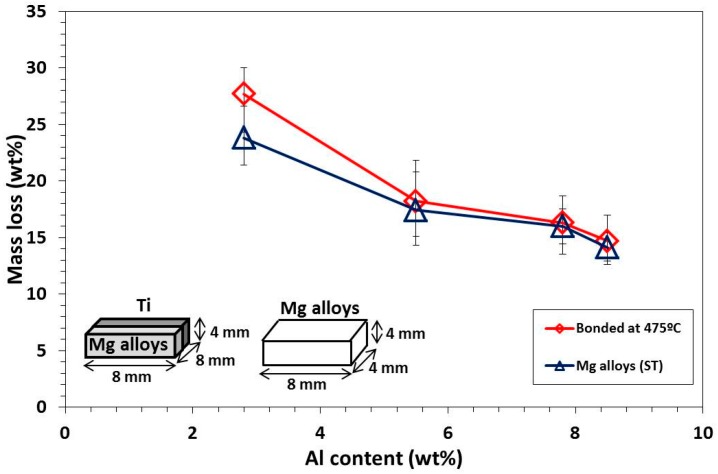
Effect of Al content on mass loss of Ti/Mg alloy (ST) bonding materials and parent Mg alloys (ST) after corrosion test in Kroll’s etchant.

Figure 10 shows a mass loss of Ti/Mg alloy (ST) dissimilar materials and Mg alloys (ST) parent metal after corrosion test in Kroll etchant for 10 min. The mass loss gradually decreased as Al content in Mg alloy (ST) increased. This was explained by the decreasing surface potential of Mg alloy when Al content increased, and the results also corresponded well to the corroded surface on Mg alloy side, in which high Al content Mg alloy showed light corrosion damage (Figure 9). The mass losses of Mg alloy (ST) parent metal were also measured after corrosion test. The mass losses of AZ31B (ST) and AZ61 (ST) parent metal were slightly lower compared to their bonding materials. This may be explained by the formation of large gap in Ti/AZ31B (ST) and Ti/AZ61 (ST) due to galvanic corrosion (Figure 9a,b). For Ti/AZ80 (ST) and Ti/AZ91 (ST), the mass losses after corrosion test were similar to their bonding materials due to smaller galvanic corrosion gaps compared to Ti/AZ31B (ST) and Ti/AZ61 (ST), which represented the good galvanic corrosion resistance of these materials. In this study, the formation of Ti_3_Al layer was found to control the tensile strength and galvanic corrosion resistance of dissimilar materials.

## 4. Conclusions

1. Ti and Mg alloys (ST) were successfully bonded with an advanced new method, spark plasma sintering without applying any inserted sheet. The surfaces of Ti and Mg alloy (ST) were perfectly contacted without any cracks or voids because of the high bonding pressure.
2. The formation of Al diffusion and Ti_3_Al layer at the nano-level were proven by controlling the bonding strength of bonding materials. A uniform and thick Ti_3_Al layer (about 50 nm) was required in order to obtain the maximum bonding strength and bonding efficiency of dissimilar materials.
3. The galvanic corrosion of dissimilar materials could be improved by formation of Ti_3_Al IMC layer. This IMC layer has a surface potential between Ti and Mg alloy, which prevents large and abrupt changes in surface potential at the bonding interface, and improves galvanic corrosion resistance
4. The mass loss of dissimilar materials was controlled by Al content in Mg alloy. The high Al content in Mg alloy contributed to a formation of a stable Mg–Al–O film, which lowers the surface potential, and resulted in high corrosion resistance on Mg alloy matrix. The galvanic corrosion may contribute to mass loss because of severe galvanic corrosion, such as in Ti/AZ31B (ST).

## Figures and Tables

**Table 1:** Chemical composition of Pure Ti rod applied in this research (wt %).

Material	Ti	Fe	H	O	N
Pure Ti	Bal.	0.3	0.013	0.13	0.05

**Table 2:** Chemical composition of cast Mg alloys rod applied in this research (wt %).

Mg Alloys	Mg	Al	Zn	Mn
AZ31B	Bal.	2.8	0.8	0.3
AZ61	Bal.	5.5	0.7	0.3
AZ80	Bal.	7.8	0.3	0.4
AZ91	Bal.	8.5	0.6	0.4
